# Three-dimensional mechanical evaluation of joint contact pressure in 12 periacetabular osteotomy patients with 10-year follow-up

**DOI:** 10.3109/17453670902947390

**Published:** 2009-04-01

**Authors:** Robert S Armiger, Mehran Armand, Kaj Tallroth, Jyri Lepistö, Simon C Mears

**Affiliations:** ^1^Johns Hopkins University Applied Physics LaboratoryLaurel, MDUSA; ^2^ORTON Orthopaedic Hospital, Invalid FoundationHelsinki, MDUSA; ^3^Department of Orthopaedic Surgery, Johns Hopkins Bayview Medical CenterBaltimore, MDUSA

## Abstract

**Background and purpose** Because of the varying structure of dysplastic hips, the optimal realignment of the joint during periacetabular osteotomy (PAO) may differ between patients. Three-dimensional (3D) mechanical and radiological analysis possibly accounts better for patient-specific morphology, and may improve and automate optimal joint realignment.

**Patients and methods** We evaluated the 10-year outcomes of 12 patients following PAO. We compared 3D mechanical analysis results to both radiological and clinical measurements. A 3D discrete-element analysis algorithm was used to calculate the pre- and postoperative contact pressure profile within the hip. Radiological angles describing the coverage of the joint were measured using a computerized approach at actual and theoretical orientations of the acetabular cup. Quantitative results were compared using postoperative clinical evaluation scores (Harris score), and patient-completed outcome surveys (q-score) done at 2 and 10 years.

**Results** The 3D mechanical analysis indicated that peak joint contact pressure was reduced by an average factor of 1.7 subsequent to PAO. Lateral coverage of the femoral head increased in all patients; however, it did not proportionally reduce the maximum contact pressure and, in 1 case, the pressure increased. This patient had the lowest 10-year q-score (70 out of 100) of the cohort. Another hip was converted to hip arthroplasty after 3 years because of increasing osteoarthritis.

**Interpretation** The 3D analysis showed that a reduction in contact pressure was theoretically possible for all patients in this cohort, but this could not be achieved in every case during surgery. While intraoperative factors may affect the actual surgical outcome, the results show that 3D contact pressure analysis is consistent with traditional PAO planning techniques (more so than 2D analysis) and may be a valuable addition to preoperative planning and intraoperative assessment of joint realignment.

## Introduction

Periacetabular osteotomy (PAO) is used to treat painful dysplasia of the hip in younger patients. The hip joint is realigned in order to theoretically stabilize the joint and reduce contact pressures (Ganz et al. 1988, Hipp et al. 1999). Achievement of optimal alignment of acetabular bone fragments is, however, reported to be the most challenging aspect of the surgery (Crockarell et al. 1999). While current navigation techniques can assist the surgeon with the technically demanding placement of osteotomes (Langlotz et al. 1998), additional means to plan and achieve ideal joint realignment are desirable.

Radiological planning for PAO involves measurement of characteristic angles of the dysplastic hip joint and comparison of these with normal angle ranges. While AP radiographs are common for identifying hip dysplasia, 3D computed tomography (CT) scans are used to evaluate early osteoarthritis, and to plan or simulate the corrective osteotomy by some surgeons (Klaue et al. 1988, Anda et al. 1991b, Tsumura et al. 2005, Tallroth and Lepisto 2006).

Discrete-element analysis (DEA) is a computationally-efficient method for modeling of cartilage stress while neglecting underlying bone stress (An et al. 1990, Genda et al. 1995, 2001, Schuind et al. 1995, Fregly et al. 2003, Pompe et al. 2003, Elias et al. 2004, Tsumura et al. 2005, Yoshida et al. 2006, Chao et al. 2007). The DEA technique models the joint contact pressure profile using linear or nonlinear compressive springs distributed over the cartilaginous region of a diarthroi-dal joint (Volokh et al. 2007). The accuracy and limitations of the technique have been evaluated using numerical and mathematical modeling techniques (Li et al. 1997) as well as cadaveric studies (Elias et al. 2004). For PAO, mechanical modeling offers a quantifiable description of the hip joint that may be realigned using computational optimization (Armand et al. 2005, Tsumura et al. 2005).

We analyzed the surgical outcomes of 12 PAO patients using mechanical, radiological, and functional analysis to show the consistency of the 3D analysis with radiological angles, and to follow-up these cases clinically.

## Methods

The study was based on retrospective evaluation of pre- and postoperative CT data of 12 consecutive PAO patients treated for symptomatic hip dysplasia under Institutional Review Board approval (JHM IRB1 #05-09-02-01). The data from the 12-patient cohort has been the basis for a previous 2-dimensional mechanical analysis (Armand et al. 2005) and for the validation of a joint contact surface segmentation technique (Armiger et al. 2007). Two independent observers performed the CT data segmentation to measure the variability of computational mechanical calculations.

## Patients

Before the inception of the present study, one of the authors (JL) completed surgeries for the 12 consecutive PAO patients between October 1995 and October 1996 at ORTON Hospital in Helsinki, Finland. Indications were based on radiologically detected hip dysplasia with several months’ history of constant pain during load bearing. Median age of the patients was 35 (20–50) years and all were female. They had neither a history of femoral head disorder (e.g. Legg-Perthes-Calvé) nor a radiographically detected deformation of the femoral head (e.g. coxa plana). Radiological evaluation was performed using both radiographs and CT, following a standard protocol (Lepistö et al. 1998) for all patients. Weight-bearing AP radiographs and CT scans were taken 1 week before the surgery and at a minimum of 4 months postoperatively.

The CT imaging involved only the hip joint. Patients lay supine and were deliberately positioned on the CT table to obtain true transverse slices of the acetabulum (as confirmed with a scout view before scanning) to ensure accurate angle measurements. A radiologist (KT) measured angles describing the acetabular coverage of the femoral head on the pre-operative CT scans. The surgical team used the radiological angles to identify deficient coverage in the dysplastic hips, and to plan the corrective reorientation of the acetabular fragment. During the procedure, the surgeon monitored the fragment realignment 3-dimensionally by affixing reference wires (K-wires) to the pelvis and to the acetabular fragment to measure relative motion. The position of the realigned acetabular fragment was confirmed using fluoroscopy before securing the fragment (Lepistö et al. 1998).

Clinical outcomes for the 12 patients were evaluated by two scoring methods. The Harris hip score (Harris 1969) involves a physical examination assessing joint function and range of motion, and a questionnaire (q-score) including an assessment of pain (Johanson et al. 1992). An independent orthopedic surgeon performed the functional hip scoring. Preoperative patient q-scores were available for only 7 of the patients, although all presented with pain (indication for PAO). The median clinical follow-up was 2 (1.3–2.2) years. At 9–10 years after surgery, a questionnaire was answered by 11 patients. The questionnaire was not completed by patient 5 because she had received a total hip arthroplasty 3 years after the PAO due to worsening osteoarthritis.

### Image processing of CT data

Direct evaluation of the CT data on a patient-specific basis required registration of the CT image volumes on a common coordinate system. Algorithms from commercial image processing and visualization software (Amira, TGS, Berlin, Germany) registered the normalized mutual information between pre- and postoperative scans. The basis for the rigid transformation was the nonoperative region of the pelvis. Once aligned, the postoperative image volume was orthogonally resampled without modifying the preoperative scans.

The lunate surface of the acetabulum on the operative side was used for both the radiological and mechanical computational analyses. We created a triangular-face model of this surface using a previously described method (Armiger et al. 2007) as a basis for the mechanical and radiological measurements.

### Mechanical evaluation of articular contact pressure

The discrete-element analysis (DEA) algorithm within the BGS gives the 3D pressure profile within the hip joint on a patient-specific basis. The geometry of the segmented contact surface model determines the location of uniformly distributed spring elements. Element area is the basis of spring constants in the normal (Kd) and shear (Ks) direction for each element.

Because only a single elastic material was modeled, the absolute values of the spring constants would not affect the calculations. The shear constant Ks was three orders of magnitude less than Kd to simulate the low frictional nature of cartilage (Genda et al. 2001, Yoshida et al. 2006). The DEA boundary conditions fixed the pelvis (reference body) in all 6 spatial degrees of freedom (DOF), while the femur could translate and rotate in any direction to seek equilibrium with the spring system. Loads were applied through the center of the femoral head to simulate standing (Bombelli 1997, Armand et al. 2005) and the peak forces during walking and sitting down (Heller et al. 2001).

The parameters calculated in the mechanical analysis included metrics about both the location and magnitude of the peak contact pressure. The location of the peak pressure was described using the center of pressure (CP) ratio. Peak pressure located laterally resulted in a CP ratio close to 0, while peak contact pressure located medially resulted in a CP ratio close to 1 (Armand et al. 2005). Additionally, the contact area was calculated based on how much of the loaded cartilage within the joint was compressively loaded. Finally, the RU angle was calculated to describe the quasi-displacement of the femoral head (U) relative to the applied joint loading vector (R) in the mechanical model. The “virtual displacement” vector was first introduced by An et al. (1990) within the context of DEA to describe joint stability based on the relative orientation of these two vector quantities.

### Optimization

In this investigation, the acetabular contact surface for each patient was optimized to determine the acetabular fragment orientation corresponding to the minimum contact pressure. Using a built-in optimization routine for nonlinear systems (lsqnonlin; MATLAB Optimization Toolbox), the computer program optimized the fragment orientation for the standing joint loading vector with an objective function that minimizes the peak contact pressure within the joint.

Optimization search limits prevented non-physiological orientation of the acetabular fragment. The maximum rotations were ± 45° in the sagittal plane, ± 60° in the frontal plane, and ± 45° in the transverse plane. The position of the acetabular fragment was held constant in translation to maintain the macroscopic mechanical state of the joint. The optimization process terminated when no further improvements to the peak pressure could be achieved (i.e. the change in peak pressure between subsequent optimization steps was less than 1e^-15^ MPa).

### Calculation of radiological angles

**Figure 1.**
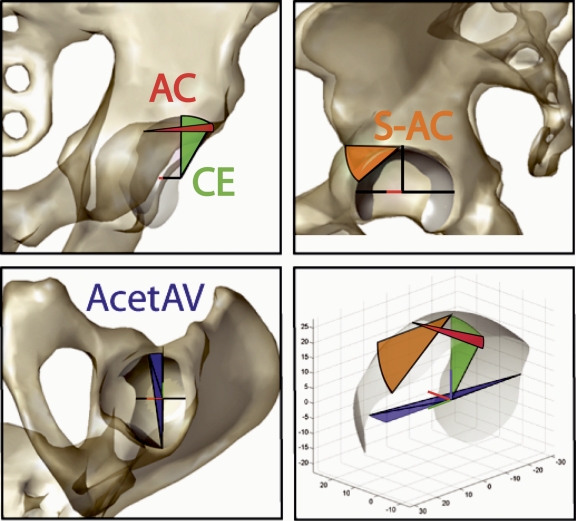
Angles describing the orientation of the acetabulum and femoral coverage are calculated from a 3D segmented model of the pelvis in frontal, sagittal, and transverse planes emulating those traditionally measured from radiographs or reformatted CT scans. In the frontal plane (top left), the AC angle measures the obliqueness of the acetabular roof between the medial aspect of the sourcil, the lateral edge of the acetabular rim, and a horizontal line. The CE angle measures the coverage of the lateral edge of the acetabulum with respect to the center of the femoral head (not shown), and may have a negative value in severely dysplastic cases. The orientation of the acetabular cup (S-AC angle) in the sagittal plane (top right) is measured from the most superior aspect of the acetabular roof to the most anterior aspect with respect to horizontal. The anteversion (AcetAV angle) is measured on a transverse plane (bottom left) viewed inferior to superior using a line created between the posterior and anterior rim of the acetabulum. The angle is measured with respect to a line normal to the two femoral head centers (intercapital centerline). The angle measurements help to characterize the orientation of the load-bearing surface of the hip joint in 3 dimensions (bottom right) during realignment (axis scale in mm; viewed isometric from anterio-lateral-superior).

Using the segmented contact surface and the computational approach, a computer algorithm was used to evaluate the radiological angles of the hip joint in the recorded pre- and postoperative configurations, and in the theoretical, optimized configuration. The characteristic angles of the hip were the CE (Wiberg 1939), AC, S-AC (Tallroth 2005), and AcetAV (Anda et al. 1991a, b, Tallroth 2005), which reflect the orientation of the acetabulum in all 3 anatomical planes (Figure 1).

Because of the limited number of datasets, we have not formed any statistical hypotheses but have looked solely at the trends in the data. The quantitative results were compared to patient-completed outcome surveys (q-score) and functional Harris score.

## Results

Between 2 independent observers, the peak pressure calculations differed by an average of 15% preoperatively; however, the discrepancy was reduced to an average absolute difference of 7.6% during the postoperative assessment, and the optimized pressures agreed within 3.4%. Calculated effective contact area followed a similar trend with a larger preoperative observer discrepancy (13%) compared to the postoperative and optimized values (2.2% and 1.7%, respectively). The computer-calculated radiological angles measured according to the contact surface model created by the 2 observers differed by 5.6 degrees.

### PAO changes in radiological angles

Using radiological measurements from the 3D contact surface, the PAO increased the lateral coverage of the femoral head in all patients (Table 1). The median increase in CE was 23° (11°–44°). Acetabular inclination (AC) decreased by 23° (10°–44°). Anterior coverage, as appraised by the S-AC angle, increased by 12° (-4°–40°) . The median change in the ante-version of the hip was found to be less than 1° (-29°–13°) degrees, based on the AcetAV angle on the transverse plane at the level of the femoral head center.

**Table 1.** Clinical results for the 12 patients

Patient no.	1	2	3	4	5	6	7	8	9	10	11	12
*q-score*												
preoperatively	76	75	62	75	–	–	68	–	74	–	–	51
2-year	99	99	68	100	45	90	86	100	–	86	–	99
10-year	100	86	100	97	–	100	100	100	70	83	98	100
*Harris:*												
2-year	100	100	100	100	52	96	96	100	100	97	100	100

### Clinical evaluation scores

The Harris score was clinically evaluated at 4 months postoperatively and 11 of the 12 patients had excellent results (Armand et al. 2005). Patient 9 scored 52 points (poor) and later had an arthroplasty. Radiographs showed that the anterior aspect of the joint had been injured during the PAO.

At 2 years, 6 patients achieved increased scores with a median increase in score of 24 (6–48) points (Armand et al. 2005). At 10 years, however, 2 patients showed declining results and the lowest score was 70 (patient 9) (Table 2).

**Table 2.** Radiological results for the 12 patients. The radiological angles associated with planning of the PAO are listed, including the center–edge (CE), acetabular inclination (AC), superior–anterior angle (S–AC) and acetabular anteversion (AcetAV)

Patient no.	1	2	3	4	5	6	7	8	9	10	11	12
*CE angle (°)*												
preoperatively	14	10	14	6	21	6	2	–6	19	14	15	3
postoperatively	24	30	25	23	47	27	23	35	42	36	35	24
optimum	26	27	26	27	38	25	24	22	26	25	30	27
*AC angle (°)*												
preoperatively	24	24	21	32	21	26	21	42	8	16	22	34
postoperatively	13	3	11	6	–13	–2	4	5	–11	1	–1	7
optimum	8	5	8	12	3	2	1	3	3	5	4	2
*S-AC angle (°)*												
preoperatively	–26	–25	–35	–34	–33	–9	–6	N **^a^**	–28	–23	–26	–1
postoperatively	–30	–32	–33	–43	–37	–38	–38	–26	–31	–31	–30	–24
optimum	–34	–35	–37	–34	–40	–31	–32	–23	–34	–26	–35	–31
*AcetAV (°)*												
preoperatively	28	19	27	20	20	26	18	29	16	17	29	38
postoperatively	27	11	30	N	N	1	6	N	25	15	N	N
Optimum	16	14	30	N	N	19	17	N	11	18	25	N

**^a^** N = Not a number; angle undefined.

### Preoperative mechanics for dysplastic patients

**Figure 2.**
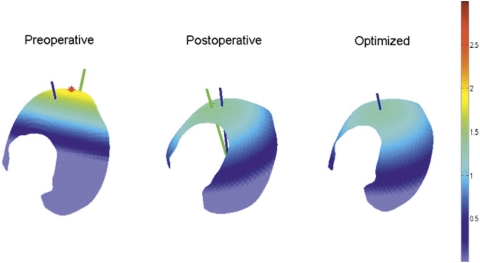
Mechanical results in which the postoperative outcome matched closely with the optimum contact pressure profile (patient 11). The blue line shown passing through the joint contact surface represents the force applied through the hip, while the green line represents the displacement direction of the femoral head. In the optimized case, the lines are collinear. Scale units are MPa.

The mechanical pressure calculations in all the preoperative cases identified peak pressure in the superior and lateral region of the acetabulum for the standing load vector (Figure 2). In the dysplastic cases, calculated peak pressures averaged 3.6 (SD 1.7) MPa. Maximum pressure was 7.8 MPa (patient 8). The virtual displacement vector was outside the articular surface in all but 1 of the preoperative cases (patient 9) and the angle between the applied joint force and the virtual displacement vector was calculated; the mean separation was 45 (SD 15) degrees.

### Mechanical evaluation following PAO

In the postoperative PAO patient group, a ratio of the peak pressure compared to the preoperative results showed the change in peak pressure due to the PAO. The PAO reduced the peak pressure by a median factor of 1.7 (0.95–2.5), and increased contact area by a factor of 1.4 (1.1–2.8). Contact area within the joint increased in all patients; however, in patient 9, the peak pressure increased by 5% postoperatively from the PAO, which is an undesirable outcome. The increase occurred despite a reduction in the magnitude of the joint force (by 100 N) due to medialization of the center of the joint. The angular direction of the joint force was unchanged (less than 0.5 degrees). The angle between the joint force and the virtual displacement vector increased for patient 9, while in all other cases this mechanical parameter decreased (Table 3).

**Table 3.** Biomechanical and radiological results for the 12 patients. The peak pressure and contact area of the acetabulum are listed. Additional parameters include the centroid of pressure (CP) and the force-displacement separation angle (RU)

Patient no.	1	2	3	4	5	6	7	8	9	10	11	12
*Pressure (MPa)*												
preoperatively	2.9	2.8	4.1	2.6	2.1	5.0	5.1	7.7	2.0	2.3	2.0	4.5
postoperatively	2.1	1.6	3.1	1.5	1.8	2.1	2.1	3.2	2.1	1.9	1.5	1.5
optimum	1.9	1.6	3.1	1.4	1.3	2.0	2.0	1.6	1.8	1.7	1.3	1.4
*Area (mm^2^)*												
preoperatively	1562	1678	1482	1937	2153	1175	1234	535	1920	1836	1984	1207
postoperatively	1824	2367	1677	2826	2861	2260	2395	1496	2370	2647	2631	2689
optimum	2144	2390	1821	2739	2904	2242	2395	1744	2255	2351	2741	2845
*CP ratio*												
preoperatively	0.12	0.03	0.02	0.02	0.03	0.05	0.01	0.12	0.26	0.02	0.03	0.09
postoperatively	0.65	0.74	0.55	0.56	1.02	0.79	0.68	1.12	1.12	0.84	0.92	0.58
optimum	0.63	0.69	0.70	0.60	0.64	0.82	0.83	0.70	0.71	0.70	0.68	0.71
*RU angle (°)*												
preoperatively	36	45	33	45	37	59	60	72	20	35	39	61
postoperatively	13	5	4	7	34	7	5	50	24	13	24	13
optimum	0	0	0	1	0	1	0	0	0	1	1	0

**Figure 3.**
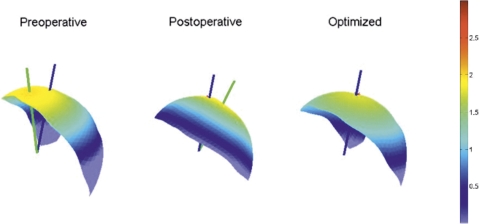
An example PAO case in which the postoperative pressure profile indicates overcorrection of the bone fragment (patient 5). The preoperative case shows pressure concentration on the lateral aspect of the joint contact surface. Excessive adduction of the acetabular fragment resulted in medially concentrated pressure postoperatively. Units are MPa.

Patient 5 was revised to a total hip arthroplasty after 3 years following osteotomy due to worsening osteoarthritis. The postoperative contact pressure profile showed overcorrection of the joint alignment, resulting in medial concentration of the peak contact pressure (Figure 3).

### Optimization of fragment orientation

We used an optimization algorithm to identify a fragment orientation that results in the minimum contact pressure (while holding the joint center and joint force constant). However, in some cases, the surgeon achieved pressures that were lower than the optimization method. The reason for this is that medialization of the joint center can reduce the joint force by changing the equilibrium between muscle forces and joint loading. This factor is independent of the joint orientation and was not considered as part of our analysis. The postoperative peak pressures achieved by the surgeon were within 0.28 (SD 0.44) MPa of the computer-optimized value. The optimized position occurred when the joint force, the peak pressure, and the virtual displacement vector were approximately coincident. Thus, the separation angle was less than 1 degree in all 12 cases after optimization.

## Discussion

In this paper we present an extended follow-up of 12 patients who underwent periacetabular osteotomy. In a previous publication (Armand et al. 2005), we presented a 2-year follow-up of these patients including clinical, radiological, and 2-dimensional mechanical evaluations. The present study implements improved mechanical and radiological analysis in addition to a longer-term (10-year) clinical follow-up. We have found no previous studies using 3D mechanical analysis for PAO on both pre- and post-operative CT data.

Comparison of the results of this 3D mechanical analysis to those of the previous 2D analysis of the same patients showed some obvious improvements in the modeling technique. The magnitudes of peak pressure calculated using the 3D contact surface ranged from 1.9 to 7.7 MPa in the preoperative cases and from 1.4 to 3.2 MPa postoperatively. These peak pressure values have the same magnitude as those reported in similar 3D contact studies of the hip (Tsumura et al. 2005, Yoshida et al. 2006). Using only the 2D geometry, a much wider and unrealistic range of pressures (3–128 MPa preoperatively and 2–34 MPa postoperatively) was found in the same 12 patients (Armand et al. 2005). Secondly, in the previous 2D study, centroid of pressure (CP) ratios within the range 0.4–0.6 were considered to be ideal; however, optimization of the entire 3D geometry of the hip resulted in an optimal range of CP between 0.6 and 0.85 (slightly medial).

Following surgery, the PAO reduced peak pressure in all but 1 case, while lateral coverage increased in every patient. Over-correction (increasing the lateral coverage beyond the normal CE range of 33 (SD 10) degrees (Janzen et al. 1998) results in negative AC angles and peak pressure located at the medial aspect of the joint (Armand et al. 2005). In this 3D study, the mechanical consequences of overcorrection were not as severe as shown in the 2D study. Only patient 9 showed an increase in pressure, by 5%. The anterior and posterior horns of the contact surface, not seen in the 2D model, contributed to the final mechanical outcome to mitigate an exponential rise in pressure with increased abduction of the acetabular cup. This suggests that the most important consequence of overcorrection of the acetabular fragment orientation is not the peak pressure, but a different factor not included in this model (e.g. impingement). An exponential rise in pressures corresponded to reduced lateral coverage of the femoral head, most likely due to the exclusion of the soft tissue labrum in this model.

An et al. (1990) discussed 3 physiological cases in the context of the mechanical model regarding the resultant force vector (R) of loading through the joint and the “virtual displacement” vector (U): (1) a stable joint is one in which both R and U pass through the articular surface; (2) joint subluxation is indicated when only R passes through the articular surface and the vector U is outside the joint space; and (3) joint dislocation occurs when both R and U are directed outside the articular surface. In the present investigation, the preoperative analyses showed 11 of the 12 patients with the U vector directed outside and lateral to the articular surface (i.e. at risk of subluxation). Postoperative analysis showed all 12 hips corrected to stable joint conditions according to the hypothesized “virtual displacement” criteria. The angle between these 2 vectors (RU angle) is an additional mechanical parameter that indicates the quality of the joint alignment.

For optimization, the objective was to minimize the peak pressure. Previous studies have shown that a minimum peak pressure exists by varying the orientation of the acetabular fragment (Tsumura et al. 2005). The contact area parameter was not sensitive enough to be a basis for planning PAO through optimization. Contact area increased in all 12 PAO cases, despite an increase in peak pressure and overcorrection of the radiological angles in one patient. The optimization routine minimizing peak contact pressure produced stable results and was evident because of the collinear (less than 1 degree) vector orientations of the peak pressure and virtual displacement. Compared radiologically, the optimized cases had CE angles in the normal range and the AC angles ranged from 1 to 12 degrees, which is consistent with the PAO surgical plan. The optimized CP ratio in this 3D study was greater (more medial) than that of the previous 2D mechanical analysis owing to the inclusion of the anterior and posterior horns in the complete 3D model. The optimization results were consistent with normal anatomy and the traditional preoperative aims of PAO. Planning of accurate correction in acetabular reorientation on 2D radiographs seems to pose limitations. Our results suggest that 3D evaluation of mechanical conditions greatly improves preoperative planning and has the potential to improve finding the right orientation for joint surfaces during surgery.

A limiting factor of this mechanical model is the calculation of the joint force, which predicates the pressure profile obtained using the DEA technique. Other optimization routines minimize pressure for only a single joint force load (Tsumura et al. 2005) while this study optimized for 3 different joint forces simultaneously. However, the in vivo dataset of joint forces (measured with an instrumented endoprosthesis (Heller et al. 2001)) has an unknown correlation to dysplastic patients, since that group included normal patients that had undergone hip arthroplasty. In addition, to apply these data correctly, the joint force must be applied using the same coordinate system as defined when recording data. The L5-S1 junction, used as a landmark in defining the local pelvis coordinate system, is not usually visible in preoperative PAO CT scans. Because patients are scanned in supine (non-weight-bearing) position, an accurate frame for mechanical analysis is not available and must be assumed.

Interobserver variability was greater in the preoperative cases than in the postoperative and optimized cases. The main factor contributing to this effect is discrepancies between observers during segmentation of the joint contact region. Variance during segmentation can affect the shape of the contact surface and this in turn can lead to variance within subsequent mechanical analyses. The effect is amplified when the joint is in an unstable condition due to dysplasia (preoperative) whereby changes to the joint surface result in large changes in contact pressure. The effects of interobserver variability are less pronounced when the joint is stable in either the postoperative and optimum configuration.

The DEA technique has been experimentally evaluated in cadavers, focusing on trends rather than absolute pressure magnitudes (Elias et al. 2004). Validation studies in dysplastic hips are not feasible due to the limited availability of appropriate specimens, and limitations of experimental models. To offset the effect of unknown absolute correlation, the mechanical results presented here reflect the ratio of pressure, rather than the absolute magnitude of pressure results, in the pre- to postoperative comparisons. A limitation associated with the use of CT data was that the cartilage tissue was not directly visible, and hence this model involved segmenting the sub-chondral bone of the pelvis and used uniform cartilage thickness, though thickness varies in both normal and dysplastic hips (Nishii et al. 2004).

Comparison of mechanical and radiological results to clinical outcomes directly using statistical methods would require more patients. Little variability existed between the 12 cases, 11 of which had excellent Harris score results. 1 patient with a poor outcome had radiological evidence of disruption of the anterior aspect of the load-bearing surface of the joint following PAO, which may have contributed to the low score. Studies on more cases with the present 3D model are needed in order to better understand the correlation between clinical outcome and the mechanical parameters described. Based on the mechanical model, we expected the change in contact pressure due to PAO to be related to patient outcome q-scores. That is, achieving the minimum contact pressure should give a favorable outcome whereas higher postoperative pressures would result in less favorable outcomes. Statistical power analysis based on the data characteristics observed in this study showed that 50 cases would be required to demonstrate significant correlation between contact pressure and patient outcome for a power of 0.8 and p < 0.05.

To conclude, the 3D mechanical model offers improvements over previous 2D models by including the anterior and posterior horns of the acetabular contact surface. This investigation indicates that optimization of the peak pressure criteria produces results that are consistent with normal patient anatomy and current surgical goals for the procedure. The rapid computation time of DEA and the radiological measurement methods make these tools potential applications in invoking real-time assessment tools for surgery.
